# Magnetic resonance imaging-guided vacuum-assisted breast biopsy: experience and preliminary results of 205 procedures

**DOI:** 10.1590/0100-3984.2017.0132

**Published:** 2018

**Authors:** Gracy de Almeida Coutinho Carneiro, Fernanda Philadelpho Arantes Pereira, Flávia Paiva Proença Lobo Lopes, Maria Julia Gregorio Calas

**Affiliations:** 1 Clínica de Diagnóstico Por Imagem ( CDPI), Rio de Janeiro, RJ, Brazil.

**Keywords:** Image-guided biopsy/methods, Breast, Magnetic resonance imaging, Breast neoplasms/diagnosis

## Abstract

**Objective:**

To demonstrate the frequency of malignancy and histological characteristics of lesions in patients submitted to vacuum-assisted breast biopsy guided by magnetic resonance imaging (MRI).

**Materials and Methods:**

This was a retrospective study of MRI-guided vacuum-assisted breast biopsies performed between April 2008 and December 2016, in which we analyzed clinical and epidemiological data, as well as the BI-RADS classification and histopathological results. We compared nodules and non-nodular enhancements, in terms of their correlation with malignancy, using chi-square test.

**Results:**

Among 215 cases referred for MRI-guided vacuum-assisted breast biopsy, the procedure was contraindicated in 10 cases (5%) and was technically feasible in the remaining 205 (95%). Non-nodular enhancements were observed in 135 cases (66%), and nodules were observed in 70 (34%), with a mean diameter of 2.2 cm (range, 0.5-9.6 cm) and 0.97 cm (range, 0.5-2.2 cm), respectively. Of the 205 lesions analyzed, 43 (21%) were malignant, 129 (63%) were benign, and 33 (16%) were classified as high-risk lesions. The most common histological findings were invasive ductal carcinoma and, in high-risk cases, lobular neoplasia. There was no significant difference between nodules and non-nodular enhancements in terms of the rate of malignancy (*p* = 0.725).

**Conclusion:**

In our sample, the overall malignancy rate was 21%. However, to improve the assessment of these results, it is necessary to correlate them with the surgical data and with data from the follow-up of benign cases.

## INTRODUCTION

Magnetic resonance imaging (MRI) of the breast is an imaging technique that is increasingly used in clinical practice, with an established role in the diagnosis, staging, and follow-up of breast cancer^(^^1^^-^^3^^)^. Among cancer patients, MRI detects additional cancer foci in the ipsilateral breast in 6-34% and in the contralateral breast in 4-24%^(^^4^^)^. In addition, MRI is able to detect lesions that are not visible on physical examination, mammography, or ultrasound^(^^4^^,^^5^^)^. According to Liberman et al.^(^^4^^)^, MRI detects 2-8% of breast neoplasms in high-risk patients in whom mammography and physical examination are normal.

MRI of the breast presents high (90-94%) sensitivity and variable (37-72%) specificity, making it necessary to confirm the malignancy of a suspicious lesion^(^^4^^,^^6^^,^^7^^)^. When the lesion has not been identified by other methods, MRI is used, either for preoperative marking followed by surgical excision or for percutaneous biopsy^(^^8^^,^^9^^)^.

Vacuum-assisted biopsy is a safe and effective technique for the management of lesions detectable only on MRI and has excellent accuracy, even for small lesions^(^^10^^-^^12^^)^. In addition, because it is a minimally invasive method, it is an alternative to surgical biopsy, without the complications and high costs associated with surgery, especially in cases of benign lesions^(^^5^^,^^13^^)^. It also enables the placement of a titanium clip (which can then be seen with other imaging methods), with the purpose of marking the affected region, in order to facilitate the follow-up or subsequent procedures^(^^5^^)^.

Although the results of MRI-guided vacuum-assisted biopsy are well established in the international literature and consensuses, data related to the results of such procedures in Brazil are still scarce. Therefore, the objective of this study was to evaluate the histopathological results and the malignancy rate found in the lesions subjected to MRI-guided biopsy at our center.

## MATERIALS AND METHODS

We conducted a retrospective study of 190 women, in whom a total of 215 MRI-guided biopsies were requested at a private clinic. Among those, biopsies were obtained from 205 lesions that could be seen only on MRI.

We used a database of patients submitted to MRI-guided vacuum-assisted biopsy at our center between April 2008 and December 2016. Clinical, epidemiological, histopathological, and breast MRI data were analyzed. All MRI scans were classified according to the Breast Imaging Reporting and Data System (BI-RADS) criteria^(^^14^^)^ as BI-RADS 5, 4, or 3, the last being assigned to patients with high familial/genetic risk or with a current diagnosis of breast cancer. Patients were advised about the procedure, post-intervention follow-up, and possible complications. All participating patients gave written informed consent.

For information management purposes, a new database containing the patient data was created with Microsoft Excel 2000, following the method used in the research protocols. After the study had been approved by the local research ethics committee, data were collected and entered into a specific electronic form.

**Figure 1:**
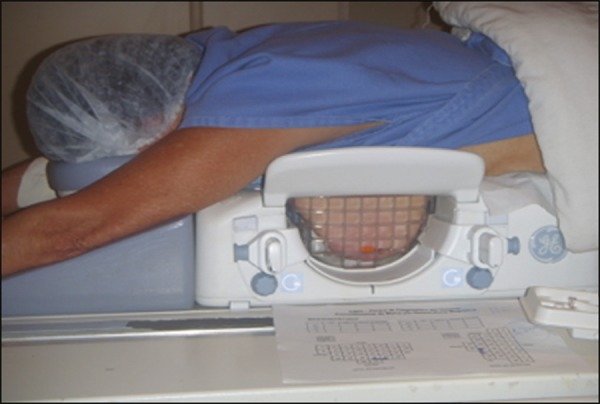
Patient positioned for the procedure, with the vitamin capsule, biopsy grid, and schematic diagram used in order to guide the biopsy.

**Figure 2:**
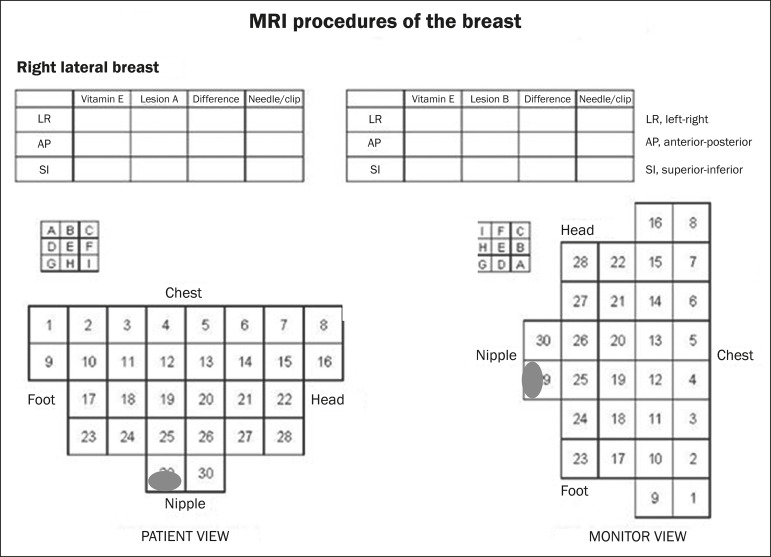
Schematic diagram used in order to guide the biopsy: in this case, lateral access to the right breast will be used.

The MRI-guided vacuum-assisted biopsies were performed by two radiologists specializing in diagnostic and interventional breast imaging. A 1.5 T MRI scanner (Signa Excite HD; GE Healthcare, Milwaukee, WI, USA) was used for the procedures. Patients were placed in the prone position, and the images were acquired with a dedicated 8-channel coil, which provides lateral and medial access to the breast (Figure 1). The target breast was compressed and immobilized. A vitamin capsule, used as marker, was placed on the skin over the region where the lesion was supposedly located, based on a review of the previous MRI study, and a schematic diagram with its location was created to be used as a guide during the procedure (Figure 2).

First, a localization sequence was acquired and the volume of interest was selected so as to include the entire compression system and the vitamin capsule. Next, sagittal fat-suppressed three-dimensional T1-weighted gradient sequences (flip angle, 15°; bandwidth, 41.67 MHz; matrix, 220 × 220; FOV, 220 mm; NEX, 1; slice thickness, 2 mm; and interslice gap, 0 mm) were acquired before and after injection via a rapid infusion pump of 0.1 mmol/L gadoterate meglumine (Dotarem; Guerbet, Roissy, France) per kilogram of body weight, followed by 20 mL of saline solution, until enhancement of the target lesion was observed.

On the monitor, the cursor was placed over the lesion. The relationship between the lesion and the surface of the skin, as well as that between the lesion and the vitamin capsule, was determined by observing sequential sagittal images. The compression grid appeared as lines with hypointense signals on the skin surface, secondary to the pressure of the grid on the skin. The vitamin capsule appeared as an oval shape, with a hyperintense signal, near the skin surface. The needle entry site was determined based on the analysis of the relationship of the lesion to the grid lines, using the vitamin capsule and the positioned cursor as guides; the skin entry site was registered in the schematic diagram (Figure 2). The depth of the lesion was calculated on the basis of the difference between the position of the slice that showed the skin surface and that of the slice that contained the lesion.

After asepsis with 70% alcohol, we anesthetized the skin surface with 5 mL of 1% lidocaine hydrochloride, without vasoconstrictors, prioritizing the needle path. We used a biopsy kit (Suros ATEC; Hologic, Marlborough, MA, USA) containing a needle guide, coaxial cannula (made of sterile plastic), stylet (made of titanium), and obturator. The needle guide was placed on the grid. The coaxial cannula, together with the stylet, was inserted until it reached the calculated depth. The stylet was then removed and replaced with the obturator, which is visible on MRI.

**Figure 3:**
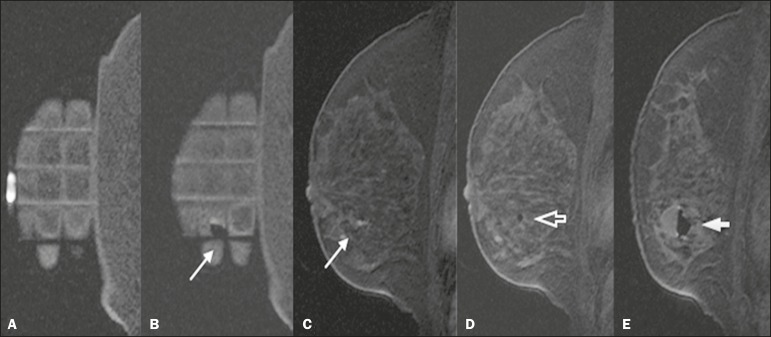
Sagittal fat-suppressed T1-weighted MRI scan of a 53-year-old woman submitted to vacuum-assisted biopsy (approach: left breast, lateral). **A**: Image of the vitamin capsule and the grid. **B**: Image of the grid and needle positioned for the procedure (arrow). **C**: Image of the lesion (arrow). **D**: Image of the obturator in the lesion (arrow). E: Post-biopsy scan showing a hematoma (arrow).

Another sagittal fat-suppressed T1-weighted sequence, with or without axial reconstruction, was then acquired to document the location of the obturator. The obturator appeared as a structure with a hypointense signal, often accompanied by an adjacent magnetic susceptibility artifact, which hindered the identification of smaller target lesions. In such cases, adjacent anatomical landmarks were useful in confirming the location of the lesion in relation to the obturator (Figure 3).

Once the obturator was correctly positioned in relation to the lesion, the patient was removed from the scanner and tissue samples were collected with the ATEC 9-gauge vacuum-assisted breast biopsy device (Hologic) through the coaxial cannula. After the samples had been collected, a titanium marker was inserted and another sagittal fat-suppressed T1-weighted sequence was acquired in order to verify the relationship between the titanium marker and the biopsied region. After the biopsy, manual compression was applied in order to ensure hemostasis. Subsequently, a sterile pressure dressing was placed over the wound and unilateral two-view mammography (in craniocaudal and lateral views) was performed to confirm the successful placement of the clip. The duration of the procedure was defined as the time from the acquisition of the localization MRI sequence to the last MRI sequence acquired, after the placement of the titanium marker. The specimens were analyzed by pathologists with experience in breast pathology.

The report containing the MRI images was given to the patients, along with their histopathological results, 15 days after the procedure. The report also included the correlation, established by the radiologist, between the biopsied image and its histopathology.

A comparative, exploratory statistical analysis was conducted using the SPSS Statistics software package, version 20.0 (IBM Corp., Armonk, NY, USA). Categorical variables are presented as absolute and relative frequencies, whereas continuous variables are presented as means and standard deviations. To distinguish nodules from non-nodular enhancements and to identify malignancy, we used Pearson’s chi-square test with a significance level of 5% (*p* = 0.05).

## RESULTS

Among a total of 215 cases referred for MRI-guided vacuum-assisted biopsy, the procedure was contraindicated in ten: because the lesion could not be identified at the time of the procedure, in six cases; because the lesion was in close proximity to the breast implant, in two; because the lesion was in close proximity to the nipple, in one; and because the lesion was located posterior to the biopsy grid, in one. Therefore, MRI-guided vacuum-assisted biopsy was performed for 205 lesions in 190 women between 26 and 85 years of age (mean age, 52 years). The proportion of technically feasible biopsies was 95%.

The most common complication of the procedure was hematoma (n = 6; 2.93%). Other complications included skin lesion (n = 1; 0.49%) and vasovagal reaction (n = 2; 0.98%). All of the complications were considered mild and self-limiting, not requiring intervention or hospitalization.

Of the 205 lesions biopsied, 135 (66%) were non-nodular enhancements and 70 (34%) were nodules. The non-nodular enhancements measured between 0.5 cm and 9.6 cm (mean, 2.2 cm), whereas the nodules measured between 0.5 cm and 2.2 cm (mean, 0.97 cm). Of the 205 lesions, 129 (63%) were benign, 43 (21%) were malignant, and 33 (16%) were high-risk lesions (encompassing nodules and non-nodular enhancements). There was no statistically significant difference between nodules and non-nodular enhancements in terms of malignancy (*p* = 0.725).

The BI-RADS lexicon was used for the findings of the pre-biopsy MRI scans. Of the 205 lesions evaluated, seven (four malignant lesions and three benign lesions) were classified as BI-RADS 5; 190 (39 malignant lesions, 119 benign lesions, and 32 high-risk lesions) were classified as BI-RADS 4; and eight (seven benign lesions and one high-risk lesion) were classified as BI-RADS 3.

The mean duration of the MRI-guided vacuum-assisted biopsy procedures performed in the present study was 26 min (range, 15-61 min), the duration varying depending on the number of target lesions and degree of difficulty of the procedure. During each procedure, we obtained no fewer than seven and no more than 20 fragments. The number of fragments obtained varied according to breast size; number, location, and size of the lesions; difficulty in performing the technique; and the presence of complications, such as bleeding and a vasovagal response.

A breakdown of the histological types of the 205 lesions can be seen in Table 1. The most common benign results were fibrocystic changes, followed by fibroadenomas, whereas the most common malignant results were ductal carcinomas, including the invasive and *in situ* forms. Among the high-risk lesions, the most common finding was lobular neoplasia.

**Table 1:** Histopathological results of the biopsies.

Histopathological result	Number of lesions
Malignant lesions	
Infiltrating ductal carcinoma	18
Ductal carcinoma *in situ*	17
Infiltrating lobular carcinoma	5
Undetermined carcinoma	2
Invasive papillary carcinoma	1
High-risk lesions	
Lobular neoplasia	11
Papilloma	8
Atypical ductal hyperplasia	6
Columnar cells changes with atypia	4
Radial scar	3
Atypical ductal hyperplasia + atypical lobular hyperplasia	1
Benign lesions	
Fibrocystic changes	77
Secretory lobular changes	19
Fibroadenoma	13
Sclerosing adenosis	9
Post-radiation changes	7
Steatonecrosis	2
Lipoma	2
Total	205

## DISCUSSION

MRI-guided vacuum-assisted biopsy is a relatively new procedure when compared with stereotactically- and ultrasound-guided biopsies. It is indicated for lesions identified only on MRI (nodules and non-nodular enhancements); that is, lesions that are not visible on other imaging methods, such as mammography, tomosynthesis and ultrasound^(^^1^^,^^5^^,^^8^^,^^15^^,^^16^^)^.

As described in the literature, the cancer detection rate of MRI-guided vacuum-assisted biopsy ranges from 17% to 61%; most studies report rates between 20% and 40%, comparable to those obtained in other types of imaging-guided biopsies^(^^4^^,^^11^^-^^13^^,^^17^^-^^26^^)^. The rate of technically feasible biopsies in our study was considered satisfactory (95%) and is in agreement with the 93-100% reported in other studies^(^^4^^,^^11^^-^^13^^,^^17^^-^^26^^)^.

In the present study, the MRI-guided vacuum-assisted biopsy was canceled in ten cases. The most common contraindication was lesion nonvisualization at the time of the biopsy, which occurred in six (2.8%) of the 215 cases. Other contraindications were lesion proximity to a breast implant (two cases) and to the nipple (one case), as well as the lesion being positioned posteriorly to the biopsy grid (one case). In the literature, a cancelation rate of 2-16% is reported for this type of procedure^(^^25^^,^^27^^)^. Lesion nonvisualization usually occurs due to differences in positioning that cause changes in the location of the lesion, compression of the breast leading to reduced blood flow to the lesion, or the procedure being performed during the incorrect phase of the menstrual cycle. These effects can be minimized by scheduling the procedure in the second or third week of the menstrual cycle, decreasing compression, and, in some cases, using delayed image acquisition^(^^4^^,^^5^^,^^8^^,^^27^^)^. In our study, the attending physician was the one who decided how to manage the ten cases in which the procedure was contraindicated. However, when in accordance with the criteria established for the BI-RADS category, surgical biopsy was recommended if the lesion could not be biopsied, whereas short-term MRI follow-up was recommended for the cases of lesion nonvisualization on the day of the procedure, as described in the literature^(^^27^^,^^28^^)^.

Because the present study was conducted at a private clinic that accepts health insurance plans, patient adherence to follow-up was not always optimal: some patients do not bring their surgery reports back to us or do not return for a follow-up examination. It is therefore difficult to define the underestimation rate of the lesions submitted to MRI-guided vacuum-assisted biopsy and the accuracy of the method. The next step of our study will be to seek follow-up data and surgical biopsy results of the patients who underwent surgery.

We compared the results of our 205 cases with those of the samples evaluated in 14 articles published between 2001 and 2016^(^^4^^,^^11^^-^^13^^,^^17^^-^^26^^)^, with lesion sample sizes ranging from 29 to 538 (Table 2).

**Table 2:** Comparison with published MRI-guided vacuum-assisted biopsy studies.

Study	Material	Number of lesions	Technical success	Duration (min.)	Cancer	High risk	Benign
Carbognin et al.^(^^17^^)^, 2011	Vacora 10-gauge	29	27/29 (93%)	9-25	11 (40%)	1 (4%)	15 (56%)
Perlet et al.^(^^12^^)^, 2002	Mammotome 11-gauge	342	334/342 (98%)	Not reported	84 (25%)	17 (5%)	233 (70%)
Perlet et al.^(^^13^^)^, 2006	Mammotome 11-gauge	538	517/538 (96%)	Not reported	138 (27%)	17 (3%)	362 (70%)
Liberman et al.^(^^4^^)^, 2003	ATEC 9-gauge	28	27/28 (96%)	35	6 (22%)	1 (4%)	20 (74%)
Liberman et al.^(^^18^^)^, 2005	ATEC 9-gauge	98	95/98 (97%)	33	24 (25%)	10 (11%)	61 (64%)
Lehman et al.^(^^19^^)^, 2005	ATEC 9-gauge	38	38/38 (100%)	38	14 (37%)	2 (38%)	22 (38%)
Orel et al.^(^^20^^)^, 2006	ATEC 9-gauge	85	85/85 (100%)	Not reported	52 (61%)	18 (21%)	15 (18%)
Gebauer et al.^(^^21^^)^, 2006	Vacora 10-gauge	44	42/44 (95%)	Not reported	11 (27%)	3 (7%)	28 (68%)
Perreta et al.^(^^11^^)^, 2008	Vacora 10-gauge	47	47/47 (100%)	Not reported	15 (32%)	4 (8%)	28 (60%)
Mahoney^(^^22^^)^, 2008	EnCor 10-gauge	55	55/55 (100%)	Not reported	10 (18%)	7 (13%)	38 (55%)
Malhaire et al.^(^^23^^)^, 2010	Vacora 10-gauge	74	72/74 (98%)	72	33 (46%)	10 (14%)	29 (40%)
Rauch et al.^(^^24^^)^, 2012	ATEC 9-gauge	218	218/218 (100%)	Not reported	48 (22%)	37 (17%)	133 (61%)
Spick et al.^(^^25^^)^, 2016	Vacora, ATEC, and Mammotome	487	487/487 (100%)	Not reported	82 (17%)	77 (16%)	328 (67%)
Ferré et al.^(^^26^^)^, 2016	SenoRx 10-gauge	259	253/259 (98%)	Not reported	93 (37%)	47 (18%)	113 (45%)
This study	ATEC 9-gauge	215	205/215 (95%)	26	43 (21%)	33 (16%)	129 (63%)

The mean duration of the MRI-guided vacuum-assisted biopsy (26 min) and the number of fragments removed during the procedure (n = 7-20), as well as the fact that there were no serious complications, were similar to the findings of the other 14 studies evaluated, especially those that used the same device and probe size as ours (ATEC, 9 gauge), and are in accordance with the international consensus on the topic^(^^16^^)^. The duration of the procedure in the present study is comparable to that of stereotactically- and ultrasound-guided vacuum-assisted biopsies, depending mostly on the number of lesions investigated and on the technical difficulties of the procedure, regardless of the localization method used^(^^20^^-^^26^^)^. We can conclude that vacuum-assisted biopsy is a safe and effective technique for the management of lesions detectable only on MRI.

Of the lesions biopsied in the present study, 63% were benign, compared with rates ranging from 38% to 74% in 13 of the 14 studies selected, the remaining study reporting a rate of 18%^(^^20^^)^. That same study reported the highest rate of malignancy (61%), most probably because it evaluated a small sample of selected cases (n = 85).

High-risk lesions accounted for 16% of the lesions in our sample, within the range of 3-21% reported in 13 of the 14 studies selected for comparison, although the remaining study reported a rate of 38%^(^^19^^)^. We found that such lesions were more common among the non-nodular enhancements.

The overall rate of malignancy found in our study was 21%, and the rate was higher (22.2%) among the non-nodular enhancements. Our data are quite similar to those reported by Liberman et al.^(^^18^^)^, Rauch et al.^(^^24^^)^, Mahoney et al.^(^^22^^)^ and Spick et al.^(^^25^^)^, although their samples differed from ours. Among our malignant cases, 17 patients (8% of the sample as a whole) had ductal carcinoma *in situ*, comparable to the 3-28% reported in the literature^(^^24^^,^^29^^,^^30^^)^. A finding of ductal carcinoma *in situ* was also more common among non-nodular enhancements than among nodules, occurring in 14 cases and three cases, respectively.

With regards to the BI-RADS classification, we observed that of the eight lesions classified as probably benign (BI-RADS 3), only one presented a high-risk histopathological result (focal atypical lobular hyperplasia), whereas the other seven were benign. Of the 190 BI-RADS 4 lesions, 39 (20%) were malignant, 32 (17%) were high-risk lesions, and 119 (63%) were benign. Of the seven BI-RADS 5 lesions, four were malignant, as expected, and three were benign-one nodule diagnosed as fibroadenoma and two lesions (one nodule and one non-nodular enhancement) diagnosed as usual ductal hyperplasia accompanied by columnar cell changes without atypia.

The BI-RADS system, with its new concepts and terminology, provides the MRI nomenclature with greater clarity and uniformity. It is known that breast MRI scans can usually provide enough information to support a recommendation for a course of action^(^^14^^)^. The malignancy rate of lesions classified as BI-RADS 3 is lower than 3%. Such lesions may be investigated in specific situations, especially in high-risk patients with a history of breast or ovarian cancer. Histological investigation is indicated for all lesions classified as BI-RADS 4 on MRI, because this category has a highly variable likelihood of malignancy (3-94%) and is not divided into the subcategories 4A, 4B and 4C (low, intermediate and high level of suspicion, respectively) for MRI, as it is for other imaging methods. BI-RADS 5 lesions present a more than 95% probability of malignancy and must also be investigated^(^^14^^)^.

Our results, notably our finding of a 57% malignancy rate among BI-RADS 5 lesions, lead us to question the BI-RADS classification used in the procedures included in our sample, because the physician who performed the procedures did not re-evaluate the BI-RADS classification. This reveals the fact that there is a necessary learning curve for professionals using the BI-RADS classification in MRI studies, highlighting the importance of having dedicated breast radiologists to perform the test. In addition, one should consider the possibility of false-negatives in MRI-guided biopsies, especially in biopsies of non-nodular lesions, because we did not have follow-up or surgical data for the patients involved.

Of the 205 patients in our sample, 76 had malignant or high-risk lesions and were appropriately referred for surgery, thus receiving proper care. Likewise, the remaining patients (those with benign results, a bit over half of the sample) were spared an unnecessary, costly surgery that would certainly have had physical and psychological consequences for their lives, which shows how impactful this procedure can be.

## CONCLUSION

MRI-guided vacuum-assisted biopsy is a method that is well tolerated, simple, safe, useful, and reproducible. However, the identification and correct classification of a lesion detectable only on MRI are critical for appropriately referring patients for biopsy. At our center, we found a 21% malignancy rate, which is in agreement with the findings of other studies in the literature. Because this was a preliminary study, studies involving the correlation with surgical data and evaluating patients over longer follow-up periods are needed in order to evaluate the true accuracy of the methods employed. To our knowledge, there are no similar data in the literature of Brazil. Therefore, our study is one of the first in this field.
